# Evaluating the Feasibility of Electronic Patient-Reported Outcomes for a Population Receiving Specific Health Checkups: A Pilot Study

**DOI:** 10.3390/healthcare14020218

**Published:** 2026-01-15

**Authors:** Hiroshi Yano, Naoki Hosogaya, Shotaro Ide, Rina Kawasaki, Tokuma Tadami, Masatoshi Ide, Kenta Murotani

**Affiliations:** 1 Clinical Research Center, Nagasaki University Hospital, Nagasaki 852-8501, Japan; 2 Department of Biostatistics, Graduate School of Medicine, Kurume University, Fukuoka 830-0011, Japan; 3 Clinical Research Support Center, Kagawa University Hospital, Kagawa 761-0793, Japan; 4 Showakai Hospital, Nagasaki 850-0911, Japan; 5 Infectious Diseases Experts Training Center, Department of Infectious Diseases, Nagasaki University Hospital, Nagasaki 852-8501, Japan; 6 Ide Internal Medicine Clinic, Nagasaki 852-8136, Japan; 7 School of Medical Technology, Kurume University, Fukuoka 830-0011, Japan; 8 Biostatistics Center, Kurume University, Fukuoka 830-0011, Japan

**Keywords:** ePRO, system usability scale, bring your own device, specific health checkups, older adults

## Abstract

**Background**: In recent years, electronic patient-reported outcome (ePRO) systems on electronic devices, such as smartphones, have been employed to collect patients’ self-assessments and symptom reports. However, these studies were limited to younger populations and patients with severe diseases. **Objective**: This study aimed to evaluate the ease of use and response continuity of an ePRO system used by healthy middle-aged and older adults. **Methods**: This prospective observational study included participants aged 40–74 years undergoing specific health checkups. The System Usability Scale (SUS) was used to assess ePRO usability. Response continuity was evaluated by assessing EuroQol 5-Dimensional 5-Level responses once a month for up to 3 months after the health checkup date. **Results**: Eleven participants, aged 47–73 years, participated in the study. The mean SUS on the screening date was 59.1 (95% CI: 50.0–68.1; a cut-off of 70 indicated “useful”). However, only one participant failed to complete the ePRO at one and two months post-examination, and responses were obtained from all participants at three months. **Conclusions**: Due to the small sample size, usability as measured by the SUS should be interpreted descriptively. While initial onboarding appeared to be a major implementation barrier, sustained monthly ePRO reporting over 3 months was achievable among participants who completed registration with support, suggesting the conditional feasibility of response continuity in this preventive health checkup setting.

## 1. Introduction

Japan is aging at an unprecedented pace, with people aged 65 years and above accounting for approximately 30% of the total population [1]. Japan has established specific health checks and guidelines for individuals aged 40–74 years as primary prevention measures. These measures aim to assist in the early detection and prevention of the severe progression of lifestyle-related diseases commonly observed among middle-aged and older adults, such as metabolic syndrome, osteoporosis, and heart disease [2].

Patient-reported outcomes (PROs) are increasingly being valued as evaluation metrics. PROs are the health status and symptom complaints reported by patients. Electronic PROs (ePROs), which collect PROs using devices, such as smartphones, offer advantages over paper forms by reducing missing data, improving data integrity through timestamps, and enabling remote monitoring. Its implementation in clinical research and healthcare settings has increased in recent years. Guidelines for implementation and best practices for transitioning from paper to electronic formats are also being systematized [3,4,5].

The use of ePROs has increased over the past decade, with accumulating reports from randomized trials and real-world clinical settings, particularly in specialized fields, such as oncology [6,7]. Some studies have demonstrated the clinical benefits of incorporating ePRO into symptom monitoring, including improved patient-clinician communication, enhanced quality of life (QoL), and prolonged survival [8]. Conversely, PRO utilization in diagnostic/screening contexts and its implementation outside severe disease surveillance remain relatively limited. In particular, the use of ePRO in population-prevention settings such as health checkups is scarce.

Access to healthcare may be further constrained by regional and situational factors [9]. Nagasaki Prefecture in Japan, where this study was conducted, comprises 41 inhabited islands [10], making travel to medical facilities challenging. Furthermore, the COVID-19 pandemic has highlighted the vulnerability of traditional in-person health examination systems and emphasized the need for alternative approaches to maintain health monitoring when face-to-face visits are restricted.

These challenges underscore the need to evaluate the practical feasibility of introducing ePROs as tools for continuous health status monitoring in preventive health checkup settings, particularly in conditions where in-person medical care is limited.

In this study, we aimed to assess the feasibility of the ePRO in a population of healthy middle-aged and older individuals who underwent specific health checkups. Ease of use, response continuity, and implementation challenges were evaluated. Demonstrating the feasibility of ePRO implementation for older populations, such as those undergoing specific health checkups, could enhance care by monitoring daily health status and the early detection of abnormalities. ePRO data may also enable information collection during pandemics, when clinic visits are restricted or when physical contact is discouraged.

## 2. Materials and Methods

### 2.1. Study Design and Participants

This prospective, observational study involved two Japanese medical institutions that conducted specific health checkups. This study evaluated the ease of use and response continuity of an ePRO among middle-aged and older individuals who underwent health checkups. The inclusion criteria were as follows: (1) Participants undergoing specific health checkups at the study institution; (2) Ages between 40 and 74 years; (3) ownership of a smartphone, possession of a personal email address for verification, and ability to complete questionnaires; and (4) Informed consent was obtained based on the full understanding of the study after receiving an adequate explanation. No exclusion criteria were established. Participants who underwent specific health checkups during the study period and met the eligibility criteria were informed of the study by a facility researcher and invited to participate. The total number of individuals who received an explanation but did not proceed to register was not recorded systematically. Therefore, this study reports the number of individuals who ultimately provided informed consent to participate.

### 2.2. ePRO System

**Figure 1:**
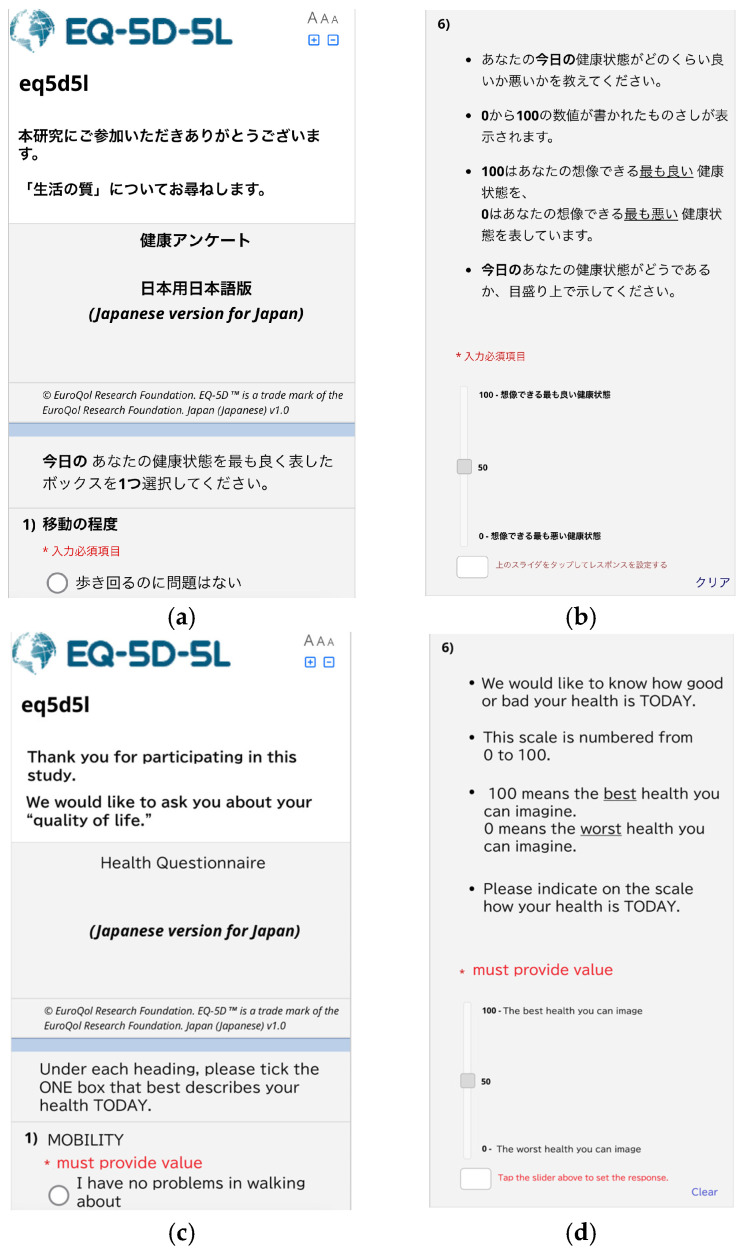
ePRO system on the device: (**a**) ePRO title and first question (single choice), (**b**) question using the Visual Analogue Scale (VAS) and (**c**,**d**) English translations of the Japanese text shown in (**a**,**b**), for reference only. * 入力必須項目: must provide value.

The ePRO system utilizes the survey functionality of the Research Electronic Data Capture (REDCap) [11], developed by Vanderbilt University. Prior to responding, participants registered their email addresses using Google Forms. The REDCap system response URL was sent to the registered email addresses. The participants opened the URL via email in their browser and completed the EuroQol 5-Dimensions 5-Level (EQ-5D-5L) [12]. Figure 1 shows the screen display after opening the ePRO system in a smartphone browser. The participants used their own smartphones to complete the responses, following the bring-your-own-device approach. Identity verification was conducted by sending the ePRO URL to the participants’ email addresses. Research collaborators at the study site provided instructions on how to use the system.

### 2.3. Usability Assessment

The System Usability Score (SUS) was calculated at the initial screening and 3 months later to assess the ease of use of the ePRO system. The SUS is a 10-item questionnaire using a 5-point Likert scale [13]. A score of 70 was considered the benchmark for good usability [14]. This study used the Japanese version of the SUS based on Yamauchi’s translation [15]. Minor wording adjustments were made to ensure clarity and participant comprehension, as required by the Ethics Committee; however, these modifications did not affect the fundamental content or scoring method of the SUS. Free-form comments regarding usability impressions were collected using questionnaires.

### 2.4. Response Continuity and Data Collection

To evaluate response continuity, the EQ-5D-5L [12,16] responses were collected at the health checkup and every month thereafter for 3 months. This pilot study evaluated the short-term feasibility and continuity of ePRO use. Therefore, the observation period was set at 3 months, based on previous research [17] on ePRO implementation. Additionally, the EQ-5D-5L responses were collected on paper only on the health checkup date and were compared with the ePRO data. REDCap was used to collect ePRO responses and study data [11].

### 2.5. Sample Size

The required sample size was 14. This was calculated based on a threshold of 70 to indicate SUS utility, an expected value of 80, a standard deviation of 15, a one-tailed significance level of 5%, and a power of 80%. Anticipating an attrition rate of approximately 40% during the 3-month observation phase, a sample size of 24 participants was calculated. The SUS threshold of 70 and expected value of 80 were referenced from prior studies evaluating ePRO [18].

### 2.6. Statistical Analysis

The primary endpoint was the SUS on the health checkup date. The mean SUS and corresponding 95% confidence intervals (CIs) were estimated using t-distribution–based methods. The internal consistency of the SUS score at each time point was assessed as a supplementary analysis; detailed results are provided in the Supplementary Material. Given the limited sample size, analyses of the SUS scores, including subgroup summaries, were conducted for descriptive and exploratory purposes only. No definitive conclusions regarding usability were made, and confidence intervals were presented solely to illustrate the uncertainty associated with this pilot sample.

Secondary endpoints included the response rate for each ePRO session, the time taken to complete the ePRO page and press the submit button, the SUS at three months, screening results, and the actual rate of follow-up visits among the cases recommended for screening. Summary statistics were calculated for each endpoint. The mean difference in SUSs between the screening visit and 3 months later was compared using a paired *t*-test. R version 4.3.3 [19] was used for statistical analysis.

## 3. Results

### 3.1. Participant Characteristics

Eleven individuals who underwent specific health checkups at two participating medical institutions, between July 2023 and October 2023, were included in this study. The participants’ characteristics are presented in Table 1. The median participant age was 67 years (range, 47–73 years). Five patients were male, and six were female. Six had a high school diploma or lower, and five had a junior college degree or higher. All 11 participants used their smartphones daily, while 45.5% (5/11) had never answered the questionnaire via a smartphone.

### 3.2. Usability Outcomes

**Figure 2:**
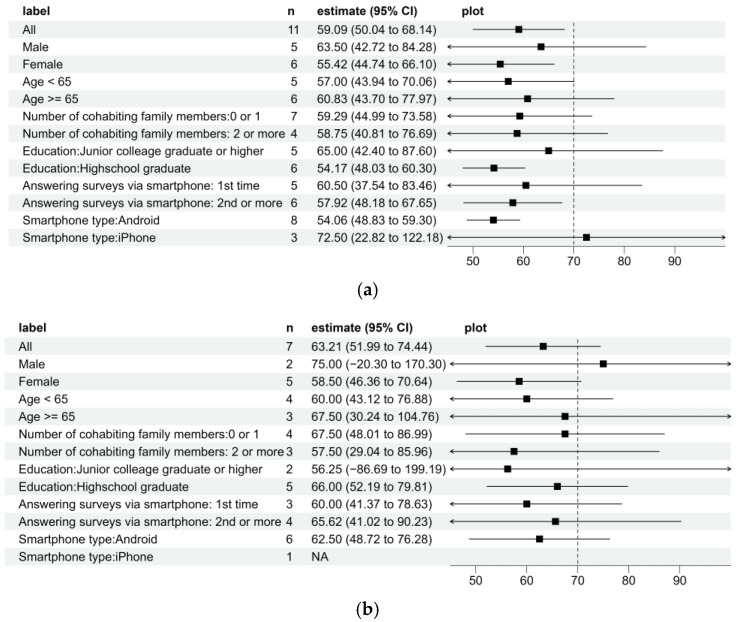
Forest plots for System Usability Scale (SUS) scores. Squares indicate point estimates, and horizontal lines represent 95% confidence intervals. The dotted vertical line indicates the target threshold of 70: (**a**) Health checkup date; (**b**) three months.

The mean SUS at the checkup date, the primary outcome measure, was 59.1 (95% CI: 50.0–68.1). As illustrated in Figure 2, the SUSs tended to be higher among iPhone users, men, and participants with higher educational levels. At 3 months, the mean SUS score increased to 63.2 (95% CI: 52.0–74.4). The paired *t*-test results showed a mean increase of 7.5 points. Owing to the small sample size, the observed mean SUS scores should not be interpreted as evidence of poor usability. Rather, they reflect substantial uncertainty and highlight the need for larger studies to properly assess usability in this population.

**Figure 3:**
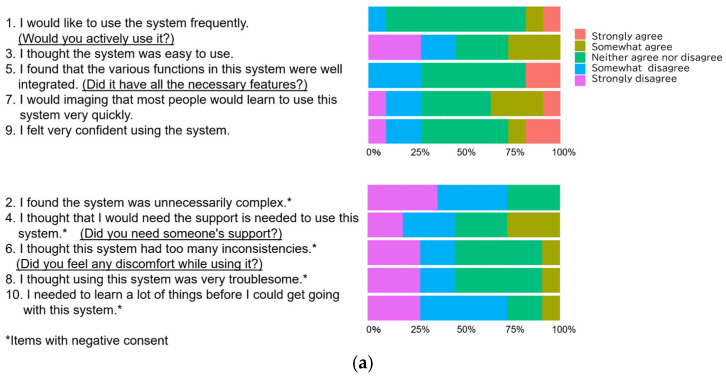

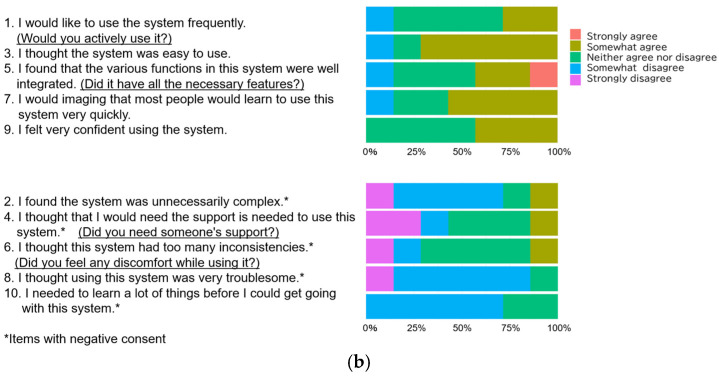
Bar charts showing responses for each SUS statement: (**a**) Health checkup date; (**b**) Three months.

The responses to each SUS question are shown in Figure 3. At the health checkup date (Figure 3a), most responses to positive SUS items were neutral or negative, and no “strongly agree” responses were observed for any negative items.

Three months after the initial health checkup, responses to positive items tended to improve, and disagreement with negative items became more frequent (Figure 3b).

Supplementary analyses of internal consistency are presented in Supplementary Table S1.

As shown in Table 2, most participants reported a similar ease of use between the ePRO and paper forms on the health checkup date. At 3 months, a greater proportion of participants perceived the ePRO as being easy to use than the paper form.

### 3.3. Continuity Results

The ePRO response rate after the checkup date remained consistently high at all follow-up points after the screening date (Table 3). Although the response time was longer at the 1-month time point, it tended to decrease with each subsequent response. Individual EQ-5D-5L responses showed minimal changes over the 3-month period following the checkup date. The data entry for the EQ-5D-5L between the paper and ePRO formats was largely consistent, with only one discrepancy identified in a single visual analog scale entry. Among the seven users with responses, only one user received a referral recommendation during screening (responded with “scheduled to visit”).

## 4. Discussion

This study introduced an ePRO system to the middle-aged and older general population undergoing specific health checkups, and evaluated its usability and response continuity. Importantly, this study should not be interpreted as demonstrating the overall feasibility of ePRO implementation for middle- and older-aged adults. Instead, our findings indicate that substantial barriers exist in the initial onboarding and registration stages, particularly in preventive health checkups. Introducing an ePRO system for middle-aged and older presents unique challenges beyond access disparities, such as age-related changes in sensory and cognitive functions and insufficient input support systems [20]. Skills in utilizing information and communication technology may also be deficient (digital divide). Previous studies have also indicated that ePROs for older users are “usable but require support.” Furthermore, the key factors enabling widespread adoption include large fonts, simple navigation, prior explanation and rehearsal, and face-to-face input support at the venue [21]. In a large observational study, Riedl et al. reported that although the overall completion rates of ePROs were high, these rates declined notably among participants aged >70 years. Importantly, the need for assistance was substantially higher when ePROs were completed at home than at supervised facilities. The authors emphasized that these findings do not reflect the intrinsic limitations of older adults, but rather the critical importance of appropriate implementation strategies and support structures. These observations are particularly relevant in preventive health checkup settings, where participants are often expected to complete ePROs independently at home after a single on-site explanation.

This study also observed the impact of the digital divide and encountered difficulties in attaining the preset target number of cases. Possible contributing factors include participants’ digital literacy (such as being unable to use an email address) and psychological resistance to device operation (including perceiving registration and procedural steps as burdensome). In addition, the SUS administered at the checkup date yielded a score of 59.8. Although this exceeded the failure threshold of 50, it was not a high score [14], suggesting the need for improvement in the current user interface/user experience (UI/UX) design, support system, and value conveyed to the participants. Given the small sample size, the SUS results should only be interpreted descriptively; no quantitative conclusions regarding usability can be drawn from this pilot study.

An important observation of this study is that continuous responses to ePRO were maintained for over 3months by all participants. These results suggest the conditional feasibility of sustained ePRO use among smartphone-owning participants who successfully completed onboarding with appropriate initial support and a user-friendly system design.

Furthermore, the SUSs showed an improvement trend after 3 months, and the time required for responses was shorter than that on the checkup date. This reduction may reflect an increasing familiarity with the ePRO process rather than improvements in system usability. Given the exploratory nature of this pilot study and the limited sample size, changes in the SUS scores and differences across participant attributes were not interpreted. Within this selected sample of smartphone-owning participants who completed onboarding, the findings suggest that middle-aged and older adults may be able to engage with digital technologies; However, this observation should not be generalized to individuals with limited digital access or lower levels of digital literacy.

Strategic interventions targeting initial operational and psychological barriers are considered key to the implementation of ePRO. Empowering the middle- and older-aged adults to overcome the digital divide cannot be accomplished overnight. However, the study’s achievement of sustained response behavior suggests that these challenges may be mitigated through appropriate implementation design and support.

The findings of this study, which clarify the challenges and potential for ePRO utilization among middle-aged and older populations in Japan, suggest that the ePRO system may be relevant for the early detection of abnormalities and timely medical visits, as a countermeasure against reduced medical visits observed during the COVID-19 pandemic [22], and for strengthening telemedicine in remote regions and depopulated areas with a high older population.

This study had several limitations. First, the sample size was small, with only 11 participants, resulting in insufficient statistical power and limited generalizability of the findings. In addition, the participants were restricted to middle-aged and older adults who owned and used smartphones. Those without devices or those uncomfortable with IT operations were excluded, thus limiting external validity. Nearly half of the participants in this study had no prior experience completing questionnaires via smartphones, highlighting substantial digital literacy barriers even among smartphone users.

Second, the observation period was limited to 3 months, which was sufficient to assess short-term response continuity but inadequate to evaluate long-term adherence. In fact, studies evaluating the improvement of quality of life and self-management capabilities in older adults using ePRO [23], as well as studies evaluating the cost-effectiveness of ePRO [24], each set an observation period of 15 months.

Third, the usability assessment relies primarily on SUS. Given the small sample size, the SUS results were interpreted descriptively, and no quantitative conclusions regarding usability could be drawn. Additionally, although the Japanese version of the SUS was based on an established translation [25], direct cross-language validation against the original English version has not been conducted, and linguistic or cultural factors may have influenced usability perceptions.

Future studies should incorporate practical refinements, such as optimizing the operation support mechanism and UI/UX according to user attributes and simplifying registration procedures. To validate our findings further, long-term studies involving multiple institutions and large sample populations are necessary.

## 5. Conclusions

This pilot study evaluated the usefulness (ease of use and response continuity) of the ePRO system among middle-aged and older health checkup recipients aged 40–74 years. Importantly, this study did not establish the overall usability or general feasibility of the ePRO systems for middle-aged and older adults. Rather, it identified initial onboarding as a major implementation barrier while suggesting the conditional feasibility of sustained ePRO reporting among smartphone-owning individuals who successfully complete registration with support. The mean SUSs, which evaluated usability on the checkup date, did not reach the target value of 70, and the target number of cases could not be achieved, suggesting a high barrier to introducing the ePRO system among healthy middle-aged and older individuals. In contrast, a certain level of response continuity was confirmed, as all participants continued to respond for up to 3 months. In addition, the response time tended to reduce over time. These results clearly indicate that implementing support measures and innovations to reduce perceived burden during initial implementation is a critical challenge that must be overcome to successfully promote and implement ePRO in the future. Practical strategies, such as the use of familiar communication platforms (e.g., messaging applications) and simplified, low-burden assessment designs delivered in small increments, may help facilitate engagement among older adults. Addressing these implementation challenges is essential for translating ePROs from pilot studies into sustainable and routine use among aging populations.

## Figures and Tables

**Table 1:** Participant characteristics.

Characteristic	Participants (N = 11)
Age (years), median (min-max)	67 (47–73)
Sex, *n* (%)	
Male	5 (45.5%)
Female	6 (54.5%)
Education, *n* (%)	
High school graduate	2 (18.2%)
Junior college/vocational school graduate	3 (27.3%)
College graduate or higher	6 (54.5%)
Number of cohabiting family members, *n* (%)	
0	1 (9.1%)
1 person	6 (54.5%)
2 or more people	4 (36.3%)
Co-residents under 45 years old, *n* (%)	
Yes	3 (27.3%)
Frequency of smartphone use, *n* (%)	
Daily	11 (100%)
Purpose of smartphone use, *n* (%)	
LINE *	9 (81.8%)
Email	8 (72.7%)
Internet	7 (63.6%)
Camera	7 (63.6%)
Experience answering surveys via smartphone, *n* (%)	
First time	5 (45.5%)
Second time or more	6 (54.5%)
Smartphone type, *n* (%)	
Android	8 (72.7%)
iPhone	3 (27.3%)

* LINE: A widely used mobile messaging service.

**Table 2:** ePRO versus paper forms and recommendations for medical examination.

Result	Checkup Date (*n* = 11)	After 3 Months (*n* = 7)
ePRO or paper forms are easier to use, *n* (%)		
Paper	2 (18.2%)	0 (0%)
Smartphone	2 (18.2%)	3 (42.9%)
About the same	7 (63.6%)	4 (57.1%)
Recommendation for medical examination, *n* (%)		
Yes (scheduled)	-	1 (14.3%)
No	-	4 (57.1%)
Unknown	-	2 (28.6%)

**Table 3:** EQ-5D-5L score and response time at each visit.

Results	Checkup Date	1 Month	2 Months	3 Months
Number of Responses, *n* (%)	10 (90.9%)	10 (90.9%)	10 (90.9%)	11 (100%)
Response Time (s) *	113.1 (65.1–161.1)	165.4 (42.9–287.9)	100.1 (62.0–138.3)	83.3 (56.1–110.4)
EQ-5D-5L Score *	0.989 (0.966–1.013)	0.966 (0.926–1.006)	0.968 (0.932–1.005)	0.981 (0.952–1.009)

* mean (95% CI).

## Data Availability

The datasets presented in this article are not readily available because time limitations. Requests to access the datasets should be directed to the corresponding author.

## Supplementary Materials

The following supporting information can be downloaded at: https://www.mdpi.com/article/10.3390/healthcare14020218/s1, Table S1: Internal consistency of the System Usability Scale (SUS) score at the checkup date and 3 months.

## Abbreviations

The following abbreviations are used in this manuscript:

ePRO	electronic Patient Reported Outcomes
EQ-5D-5L	EuroQol 5-Dimensions 5-Level
REDCap	Research Data Capture
SUS	System Usability Scale
UI/UX	User Interface, User Experience

## References

[1] Statistics Bureau of Japan *Statistical Handbook of Japan*; Statistics Bureau of Japan: Tokyo, Japan, 2024. https://www.stat.go.jp/english/data/handbook/pdf/2024all.pdf.

[2] Ministry of Health, Labour and Welfare, Japan Specific Health Checkups and Specific Health Guidance. https://www.mhlw.go.jp/english/wp/wp-hw3/dl/2-007.pdf.

[3] U.S. Food and Drug Administration (FDA) (2009). Guidance for Industry Patient-Reported Outcome Measures: Use in Medical Product Development to Support Labeling Claims. https://www.fda.gov/regulatory-information/search-fda-guidance-documents/patient-reported-outcome-measures-use-medical-product-development-support-labeling-claims.

[4] Mowlem F.D., Elash C.A., Dumais K.M., Haenel E., O’Donohoe P., Olt J., Kalpadakis-Smith A.V., James B., Balestrieri G., Becker K. (2024). Electronic Clinical Outcome Assessment Consortium. Best practices for the electronic implementation and migration of patient-reported outcome measures. Value Health.

[5] Coons S.J., Eremenco S., Lundy J.J., O’Donohoe P., O’Gorman H., Malizia W. (2015). Capturing patient-reported outcome (PRO) data electronically: The past, present, and promise of ePRO measurement in clinical trials. Patient-Patient-Centered Outcomes Res..

[6] Basch E., Deal A.M., Kris M.G., Scher H.I., Hudis C.A., Sabbatini P., Rogak L., Bennett A.V., Dueck A.C., Atkinson T.M. (2016). Symptom monitoring with patient-reported outcomes during routine cancer treatment: A randomized controlled trial. J. Clin. Oncol..

[7] Okuyama H., Takada F., Taira N., Nakamura S. (2024). A randomized trial of the impact of symptom monitoring using an electronic patient-reported outcome app on health-related quality of life in postmenopausal breast cancer patients receiving adjuvant endocrine therapy. Breast Cancer.

[8] Basch E., Deal A.M., Dueck A.C., Scher H.I., Kris M.G., Hudis C., Schrag D. (2017). Overall survival results of a trial assessing patient-reported outcomes for symptom monitoring during routine cancer treatment. JAMA.

[9] Mensah B., Goldstein L.B. (2025). The Impact of Telehealth on Access to Healthcare in Rural Communities. Int. J. Clin. Rep. Stud..

[10] Nagasaki Prefecture Regional Development Department, [Nagasaki Prefecture Remote Islands Development Plan], 2023. https://www.mlit.go.jp/kokudoseisaku/chirit/content/001619212.pdf.

[11] Harris P.A., Taylor R., Thielke R., Payne J., Gonzalez N., Conde J.G. (2009). Research electronic data capture (REDCap)—A metadata-driven methodology and workflow process for providing translational research informatics support. J. Biomed. Inform..

[12] Herdman M., Gudex C., Lloyd A., Janssen M.F., Kind P., Parkin D., Bonsel G., Badia X. (2011). Development and preliminary testing of the new five-level version of EQ-5D (EQ-5D-5L). Qual. Life Res..

[13] Brooke J. (1996). SUS: A ‘quick and dirty’ usability scale. Usabil. Eval. Ind..

[14] Bangor A., Kortum P.T., Miller J.T. (2008). An empirical evaluation of the system usability scale. Intl. J. Hum. Comput. Interact..

[15] Yamauchi S. (2015). An Introduction to Research Design Involving Human Subjects for Engineers.

[16] Ikeda S., Shiroiwa T., Igarashi A., Noto S., Fukuda T., Saito S., Shimozuma K. (2015). Developing a Japanese version of the EQ-5D-5L value set. J. Natl. Inst. Public Health.

[17] Bingham C.O., Gaich C.L., DeLozier A.M., Engstrom K.D., Naegeli A.N., De Bono S., Banerjee P., Taylor P.C. (2019). Use of daily electronic patient-reported outcome (PRO) diaries in randomized controlled trials for rheumatoid arthritis: Rationale and implementation. Trials.

[18] Lehmann J., Schreyer I., Riedl D., Tschuggnall M., Giesinger J.M., Ninkovic M., Huth M., Kroberger I., Rumpold G., Holzner B. (2022). Usability evaluation of the Computer-Based Health Evaluation System (CHES) eDiary for patients with faecal incontinence: A pilot study. BMC Med. Inform. Decis. Mak..

[19] R Core Team R: A Language and Environment for Statistical Computing.

[20] Mubarak F., Suomi R. (2022). Elderly forgotten? Digital exclusion in the information age and the rising grey digital divide. Inq. J. Health Care Organ. Provis. Financ..

[21] Riedl D., Lehmann J., Rothmund M., Dejaco D., Grote V., Fischer M.J., Rumpold G., Holzner B., Licht T. (2023). Usability of electronic patient-reported outcome measures for older patients with cancer: Secondary analysis of data from an observational single center study. J. Med. Internet Res..

[22] Hernandez J., Batio S., Lovett R.M., Wolf M.S., Bailey S.C. (2024). Missed healthcare visits during the COVID-19 pandemic: A longitudinal study. J. Prim. Care Community Health.

[23] Steele Gray C., Chau E., Tahsin F., Harvey S., Loganathan M., McKinstry B., Mercer S.W., Nie J.X., Palen T.E., Ramsay T. (2021). Assessing the implementation and effectiveness of the electronic patient-reported outcome tool for older adults with complex care needs: Mixed methods study. J. Med. Internet Res..

[24] Miranda R.N., Bhuiya A.R., Thraya Z., Hancock-Howard R., Chan B.C., Gray C.S., Wodchis W.P., Thavorn K. (2022). An electronic patient-reported outcomes tool for older adults with complex chronic conditions: Cost-utility analysis. JMIR Aging.

[25] Sato K., Mitomi N., Kon K., Haruna H. (2022). Examination of the Reliability of the System Usability Scale (SUS) in the Prosthetics and Orthotics Field. J. Jpn. Acad. Prosthetists Orthotists.

