# Supplementary material for: Evaluating the Feasibility of Electronic Patient-Reported Outcomes for a Population Receiving Specific Health Checkups: A Pilot Study
Source: Healthcare (Basel). 2026 Jan 15;14(2):218. doi: 10.3390/healthcare14020218 (PMC12840900; doi:10.3390/healthcare14020218)
Supplement: Supplementary file 1 [file healthcare-14-00218-s001.zip › healthcare-4028778-supplementary.pdf]

Supplementary Material

Evaluating the feasibility of electronic patient-reported outcomes for a population receiving specific health checkups: A pilot study

Hiroshi Yano, Naoki Hosogaya, Shotaro Ide, Rina Kawasaki, Tokuma Tadami, Masatoshi Ide and Kenta Murotani

Supplementary Methods

The internal consistency of the System Usability Scale (SUS) score was assessed at each evaluation point using Cronbach's alpha as a supplementary analysis. Negatively worded items (items 2, 4, 6, 8, 10) were reverse-scored according to the original SUS scoring procedure. Automatic key checking was not applied. Given the small sample size and exploratory nature of this pilot study, reliability estimates were interpreted descriptively.

Supplementary Table S1. Internal consistency of the System Usability Scale(SUS) score at the checkup date and 3 months

Time point	Cronbach's α*	95% CI (Feldt)
Checkup date	0.70	0.33-0.90
3 months	0.80	0.47-0.96

Supplementary Notes

Item-level analyses indicated heterogeneity in item–total correlations at both time points, which is not unexpected in a pilot study involving older adults and a small sample size. Therefore, the reliability estimates are provided for reference purposes only and should be interpreted with caution.
